# Regularity of Cardiac Rhythm as a Marker of Sleepiness in Sleep Disordered Breathing

**DOI:** 10.1371/journal.pone.0122645

**Published:** 2015-04-10

**Authors:** Marc Guaita, Umberto Melia, Montserrat Vallverdú, Pere Caminal, Isabel Vilaseca, Josep M. Montserrat, Carles Gaig, Manel Salamero, Joan Santamaria

**Affiliations:** 1 Multidisciplinary Unit of Sleep Disorders, Hospital Clinic, Barcelona, Spain; 2 Institut d’Investigacions Biomèdiques August Pi i Sunyer (IDIBAPS), Barcelona, Spain; 3 Dept. ESAII, Centre for Biomedical Engineering Research, BarcelonaTech, CIBER of Bioengineering, Biomaterials and Nanomedicine (CIBER-BBN), Barcelona, Spain; 4 Department of Otorhinolaryngology, Hospital Clinic, Barcelona, Spain; 5 Ciber Enfermedades Respiratorias (CIBERES), Madrid, Spain; 6 Medical School, University of Barcelona, Barcelona, Spain; 7 Department of Pneumology, Hospital Clinic, Barcelona, Spain; 8 Department of Neurology, Hospital Clinic, Barcelona, Spain; 9 Ciber Enfermedades Neurológicas (CIBERNED), Barcelona, Spain; 10 Department of Psychiatry, Hospital Clinic, Barcelona, Spain; Université de Montréal, CANADA

## Abstract

**Aim:**

The present study aimed to analyse the autonomic nervous system activity using heart rate variability (HRV) to detect sleep disordered breathing (SDB) patients with and without excessive daytime sleepiness (EDS) before sleep onset.

**Methods:**

Two groups of 20 patients with different levels of daytime sleepiness -sleepy group, SG; alert group, AG- were selected consecutively from a Maintenance of Wakefulness Test (MWT) and Multiple Sleep Latency Test (MSLT) research protocol. The first waking 3-min window of RR signal at the beginning of each nap test was considered for the analysis. HRV was measured with traditional linear measures and with time-frequency representations. Non-linear measures -correntropy, CORR; auto-mutual-information function, AMIF- were used to describe the regularity of the RR rhythm. Statistical analysis was performed with non-parametric tests.

**Results:**

Non-linear dynamic of the RR rhythm was more regular in the SG than in the AG during the first wakefulness period of MSLT, but not during MWT. AMIF (in high-frequency and in Total band) and CORR (in Total band) yielded sensitivity > 70%, specificity >75% and an area under ROC curve > 0.80 in classifying SG and AG patients.

**Conclusion:**

The regularity of the RR rhythm measured at the beginning of the MSLT could be used to detect SDB patients with and without EDS before the appearance of sleep onset.

## Introduction

Sleep-disordered breathing (SDB) is a common disorder with a range of harmful sequelae [1]. One of the most important symptoms is excessive daytime sleepiness (EDS) which has been related to an increase of driving accidents, psychosocial morbidity and poor quality of life [2–4]. Despite its relevance in clinical management, evaluation of EDS is hindered by the lack of a simple objective method.

Subjective sleepiness scales are easy to fill out but correlate poorly with objective measures [5, 6] because patients sometimes are unaware of their sleepiness or it is confounded with fatigue or depression [7]. In contrast, the multiple sleep latency test (MSLT) [8] and the maintenance of wakefulness test (MWT) [9] which are accepted as the gold standards to objectively assess EDS, are relatively complex and expensive to perform on daily routine. Thus, there is a pressing need to develop simplified objective methods that could be used broadly in clinical and real-life scenarios.

Recent studies suggest that changes in the level of sleepiness are associated with changes in autonomic nervous system (ANS) activity [10, 11]. For instance, somnolent SDB patients have an abnormal sympatho-vagal balance during sleep [11] and an increased sympathetic tone during daytime wakefulness that normalizes after continuous positive airway pressure (CPAP) treatment [12]. This suggests that the structural alterations and dysfunction in central autonomic regulatory regions occurring in SDB might contribute to EDS [13].

In this context, ANS activity could be a potential candidate to measure EDS in SDB. The simplest way to monitor ANS activity is by measuring the heart rate variability (HRV), which describes fluctuations in autonomic inputs to the heart over time. It is measured by the variation in the beat-to-beat (RR) interval in the electrocardiogram (EKG) [14]. Different methods have quantified HRV. From the traditional linear measures to the more sophisticated time-frequency representation and non-linear techniques.

Mean heart rate (HR), a simple time-domain measure, gradually decreases as sleep begins and achieves its lower value when stable N2 sleep stage appears [15–18]. In preadolescents a significant decreasing in heart rate even occurs as earlier as 30 seconds before the appearance of stage N1 [15]. It has also been described that subjects with longer sleep latencies in the MSLT and MWT present an increased HR at the beginning of each test [19–21]. Moreover, using some frequency-domain measures, Bonnet et Arand also found an increased sympatho-vagal balance in the non-sleepy subjects, without changes in the parasympathetic nervous system activity [22]. These findings in healthy adults suggest that measurements of ANS activity during wakefulness periods could help to study the EDS associated to SDB.

Non-linear methods have been developed recently to describe non-linear fluctuations in heart rate and inform about the regularity of heart rate time series [23]. It has been reported that non-linear dynamics of EEG signal during the first wakefulness period at the beginning of the MSLT is more regular (i.e. lower complexity) in SDB patients with objective EDS than in those without EDS [24]. However, little is known about the non-linear dynamics of cardiac activity related to EDS.

Using HRV measures, we aimed to find possible markers of ANS activity that could anticipate sleep onset in SDB patients and, therefore, detect patients with and without objective EDS. We analysed the first 3-min waking periods of the MWT and the MSLT to perform the study.

## Materials and Methods

### Subjects

From a series of 98 consecutive patients with suspected SDB evaluated at the Multidisciplinary Sleep Disorders Unit of the Hospital Clinic of Barcelona, two groups of 20 consecutive patients each were selected, based on mean sleep latencies from a MWT-MSLT research protocol. The sleepy group (SG) consisted of the most somnolent patients who have both low MSLT (<8 min) and low MWT (<20 min) sleep latencies while the alert group (AG) represented the least somnolent patients with the higher MWT (≥ 20 min) and MSLT (≥ 8 min) sleep latencies. Patients with discordance between MWT and MSLT scores (patients with MWT ≥20 min and MSLT <8 min or MWT <20 min and MSLT ≥8 min) were considered partially sleepy and were not included in the analysis. Exclusion criteria were age under 18 years, major medical or psychiatric disorders, use of beta-blockers or medications affecting wakefulness or sleep, and working in shifts or with irregular sleep-wake schedules during the four weeks before the sleep study. Nocturnal polisomnography (PSG) excluded any concomitant sleep disorder other than SDB.

The study was approved by the Hospital Clinic of Barcelona ethics committee (Comité Ètic Investigació Clínica (CEIC)) and written informed consent was obtained from all participants.

### Design

Patients arrived to the sleep lab at 6 pm and underwent a 24-hour sleep study. Subjective daytime sleepiness and mood disorders were assessed using the Epworth Sleepiness Scale and the Hospital Anxiety and Depression Scale. After nocturnal PSG, a MWT-MSLT research protocol was conducted to quantify EDS throughout the day. An overview of the protocol is shown in Table 1.

**Table 1:** Sleep Study Design.

**18:00**	**Enter to Sleep Lab**
**18:30**	**ESS, HAD**
**19:00**	**Electrodes placement**
**20:00**	***Dinner***
**23:00**	**Start PSG**
**7:30**	**End PSG**
**7:30 – 8:15**	***Breakfast***
**8:30 – 9:10**	**1** ^**st**^ **MWT**
**9:30 – 9:50**	**1** ^**st**^ **MSLT**
**10:30 – 11:10**	**2** ^**nd**^ **MWT**
**11:30 – 11:50**	**2** ^**nd**^ **MSLT**
**12:30 – 13:10**	**3** ^**rd**^ **MWT**
**13:30 – 13:50**	**3** ^**rd**^ **MSLT**
**13:50 – 14:15**	***Lunch***
**14:30 – 15:10**	**4** ^**th**^ **MWT**
**15:30 – 15:50**	**4** ^**th**^ **MSLT**
**16:30 – 17:10**	**5** ^**th**^ **MWT**
**17:30 – 17:50**	**5** ^**th**^ **MSLT**

ESS, Epworth Sleepiness Scale; HAD, Hospital Anxiety and Depression Scale PSG, Polysomnography; MWT, Maintenance of Wakefulness Test; MSLT, Multiple Sleep Latency Test.

Nocturnal PSG was performed according to standard practice parameters and diagnostic criteria [25, 26]. We recorded EEG (O2-A1, O1-A2, C4-A1, C3-A2, F4-A1, F3-O2), electrooculography, EKG, chin and right and left anterior tibialis surface electromyography, and synchronized audiovisual recording. Cannula, thermistor, abdominal and thoracic strain gauges, and finger pulse oximeter were used to measure respiratory variables. Apnea was defined as a complete cessation of airflow, measured using thermistor, for ≥10 sec. Hypopnea was defined as ≥30% reduction in nasal pressure signal excursions from baseline and associated ≥3% desaturation from pre-event baseline or an arousal. The apnea-hypopnea index (AHI) was the number of apneas plus hypopneas per hour of sleep. Sleep stages were manually scored according to Rechtschaffen and Kales criteria using 30-s epochs.

### MSLT and MWT protocol

Patients underwent 5 trials of MWT followed by a research version MSLT [8, 27], every two hours starting at 8:30 am. We recorded in total 200 MSLT and 200 MWT naps. The order in which the tests took place was the same for all subjects. Between naps, patients were allowed to leave their rooms and stay in the waiting area, performing routine activities or interacting with other patients in a quiet way. They were advised to avoid sleep between naps and technicians ensured this. Caffeine beverages were not allowed.

For the MWT, patients were seated at 45°, and were instructed to “remain awake for as long as possible”. For the MSLT, patients were instructed to “lie quietly in a comfortable position and try to fall asleep”. Test conditions, light intensity and temperature followed the standard recommendations from AASM 2005 [28]. Additional EKG was recorded using a 2-channel bipolar monitoring system with one electrode placed 2 cm below the right clavicle and the other 2 cm below the left clavicle.

If no sleep occurred MWT and MSLT trials were ended after 40 and 20 minutes respectively, or after unequivocal sleep, defined as three consecutive epochs of stage 1 sleep, or one epoch of any other stage of sleep. Objective daytime sleepiness was measured from sleep latency defined as time from lights out to the first epoch of unequivocal sleep in each test.

### Assessment of Heart Rate Variability

The RR series, intervals between consecutive beats, were obtained from each EKG nap recording with a sampling frequency of 256 Hz. After removing artifacts and ectopic beats, RR signals were resampled at 4 Hz. Naps with sleep latencies shorter than two minutes or EKG artifacts could not be analysed and were excluded. Then, the first waking 3-min window of RR signal at the beginning of each nap test was considered for the analysis whenever possible; otherwise we decided to fix a minimum window size of 2-min.

Heart rate variability was described by measures obtained from traditional time-domain analysis (mean and standard deviation of RR interval), power spectral analysis in frequency-domain (individual low frequency and high frequency spectral power and low frequency to high frequency spectral powers ratio) [14] and Time-Frequency Representations (TFR) based on Choi-Williams Distribution [29]. Non-linear measures—correntropy (CORR) and auto-mutual-information function (AMIF)—were used to describe the regularity of the RR signal since they are suitable to be constructed based on short-term series [30, 31]. The applied methodology, the parameters involved in the calculation of TFR, CORR and AMIF are shown in the S1 File. All these measures were calculated in the following frequency bands: low frequency (LF: 0.04–0.15 Hz), high frequency (HF: 0.15–0.4 Hz) and total band (TB: total frequency band). The analysis in the very low frequency band (<0.04 Hz) was not performed because 5-min of RR signal is the minimal window recommended for this purpose [14].

Since the present study was carried out analyzing only one short-length window of RR for each nap, the stationarity does not represent a significant problem [14]. A final check by visual inspection was carried out in order to ensure the analysis of artifact-free RR epochs.

### Data and statistical analysis

Mean values of HRV measures of all MWT and all MSLT naps for each patient were considered for the analysis. They could be calculated if at least 3 MSLT and 3 MWT naps had available data.

Heart rate variability measures were compared between AG and SG using Mann-Whitney *U* test and within each group (between the MWT and the MSLT) with Wilcoxon signed-rank test. Bonferroni correction was applied and a significance level

*p*<0.004 was taken into account. Those HRV parameters that significantly differed between groups were evaluated throughout the day to confirm the results obtained in the average analysis. Associations between HRV measures and mean sleep latencies were evaluated with Spearman rank-order test, with a statistical significance assumed for *p*<0.05.

A discriminant function was built with those HRV parameters that significantly differed between groups. The leaving-one-out method was performed as a validation method. Sensitivity (*Sen*) and specificity (*Spe*) were calculated for testing the performance of the measures. The proportion of SG patients correctly classified was counted by *Sen* and the proportion of AG patients correctly classified by *Spe*. The area under the ROC curve (*AUC*) was also used to test the performance of the measures. The ROC curve was computed for the results of the predictions calculated with a logistic regression classification using a generalized linear model. The model was built by fitting a generalized linear regression of the predicted classes on the measures, using a normal distribution [32].

## Results

Patient’s characteristics and PSG results are shown in Table 2. Most patients were male and overweight. The SG was slightly younger and tended to have more subjective complaints of daytime sleepiness in comparison to the AG. All subjects slept well, with mean sleep efficiency higher than 80% and more than 6 h of sleep. Sleep structure was similar in both groups, but the longer stage 2 sleep latency in the AG. There was a wide spectrum of disease severity in both groups but the mean AHI and the associated oxygen desaturation index tended to be higher in the SG than in the AG, without achieving statistical significance. As expected by selection criteria, SG had shorter sleep latencies than the AG: MWT (11.5 ± 4.54 min *versus* 35.3 ± 6.33 min, *p*<0.001) and MSLT (4.4 ± 1.96 min *versus* 11.66 ± 2.41 min, *p*<0.001).

**Table 2:** Clinical and PSG characteristics: descriptive data and differences between groups.

		SG	AG	*p*-values
**CLINICAL VARIABLES**	**Sex (Male/Female)**	14/6	14/6	NS
	**Age (years old)**	53.4 ± 6.0	57.5 ± 7.8	*0.04
	**Body Mass Index (kg/ m** ^**2**^ **)**	29,9 ± 4,5	29,2 ± 4.8	NS (0.48)
	**Epworth Sleepiness Scale**	12.8 ± 3.9	10.9 ± 4.6	NS (0.09)
	**HADS-A**	5.9 ± 2.8	4.9 ± 3.0	NS (0.24)
	**HADS-D**	4,4 ± 3,3	2,9 ± 2.9	NS (0.09)
**SLEEP QUALITY**	**Time in Bed (min)**	463.6 ± 29.0	468.0± 28.5	NS (0.75)
	**Total Sleep Time (min)**	381,2 ± 75,7	372,5 ± 48.8	NS (0.34)
	**Sleep Efficiency (%)**	81,1 ± 14,6	79,8 ± 8.9	NS (0.15)
	**Wake After Sleep Onset (min)**	70,8 ± 54,2	69,5 ± 34.1	NS (0.36)
**SLEEP QUALITY**	**Stage 2 sleep latency (min)**	18,0 ± 23,3	25,2 ± 19.9	*0.01
	**REM sleep latency (min)**	132,1 ± 83,9	102,7 ± 67.3	NS (0.32)
	**Stage 1 (%)**	17.7 ± 10.6	17.4 ± 9.0	NS (0.98)
	**Stage 2 (%)**	59,3 ± 8,7	56,8 ± 9.2	NS (0.81)
	**Stage 3 (%)**	8.4 ± 6.7	11.4 ± 7.9	NS (0.28)
	**REM sleep (%)**	14.7 ± 6.8	14.4 ± 6.0	NS (0.86)
	**Number of REM episodes**	3,6 ± 1,6	3,7 ± 1.5	NS (0.99)
	**Number of Phase Changes**	181.5 ± 68.5	171.8 ± 58.3	NS (0.83)
	**PLM Index (events/h)**	9,0 ± 22,8	6,2 ± 11.8	NS (0.98)
**RESPIRATORY PARAMETERS**	**Arousal Index (events/h)**	39,7 ± 22,9	32,4 ± 21.1	NS (0.21)
	**Apnea-Hypopnea Index (events/h)**	40,1 ± 28,0	27,7 ± 26.9	NS (0.06)
	**Mean SaO**_**2**_ **(%)**	93,3 ± 2,1	93,2 ± 3.0	NS (0.61)
	**Cumulative time spend below a SaO**_**2**_ **of 90%**	10,5 ± 13,5	9,7 ± 16.7	NS (0.19)
	**Oxigen desaturation Index 3%**	31,1 ± 25,0	22,0 ± 29.0	NS (0.08)
	**Nadir of Sa O**_**2**_	77,8 ± 8,9	81,2 ± 12.0	NS (0.18)

With the exception of sex proportion all results are expressed as mean ± SD. Level of significance was for p<0.05. NS, non-significant. Abbreviations: SG, Sleepy Group; AG, Alert Group; HADS-A, hospital anxiety and depression scale—Anxiety; HADS-D, hospital anxiety and depression scale—Depression; PLM Index, periodic limb movements index; Sa O2, oxygen desaturation

Of the 400 naps recorded, thirty naps (7.5%) had sleep latencies shorter than 2 minutes or had EKG artefacts that did not allow interpreting the RR signal. Three subjects from the SG did not have the minimal HRV measures required (at least 3 MSLTs and 3 MWTs naps with available data) and were excluded from the analysis. Regarding the window size of the RR signal, 344 from the remaining 370 available naps (93%) were analysed using three minutes and in the other 26 out naps with latencies between 2 and 3 minutes the window size equalled the length of sleep latency.

**Fig 1:**
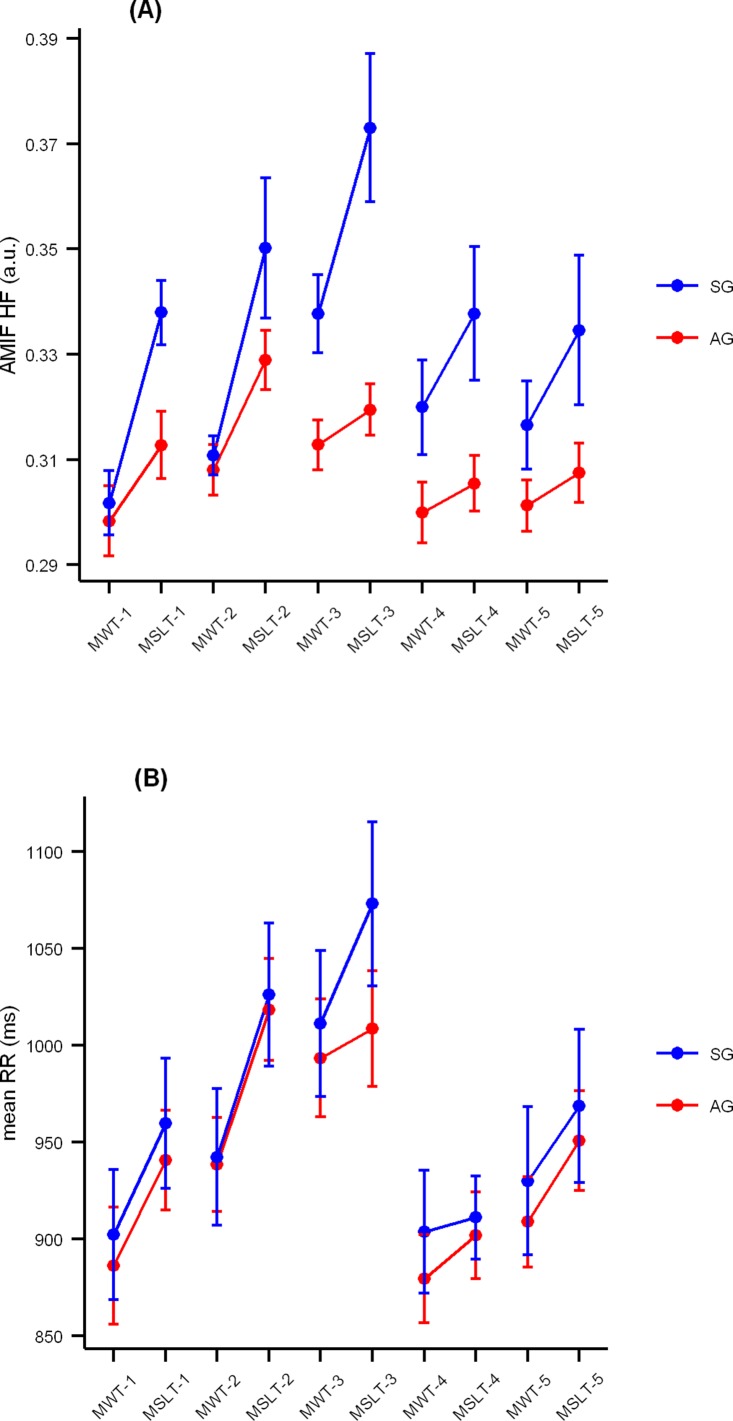
Representation of AMIF-HF and mean RR interval throughout the 5 blocks of MWT and MSLT. Sleepy group, in blue, alert group, in red. Point and error bars represent mean ± standart errors. In graphic (A), SG shows an increased regularity of RR rhythm during almost all nap tests in comparison to AG, especially at the MSLT. Within each group, however, there is a type of nap test effect, with increased values at the MSLT in comparison to MWT. In graphic (B), both groups show a reliably longer mean RR interval (i.e. slower heart rate) during MSLT as compared to MWT, but there are no differences between groups in any test. The lowest values of all naps are seen during the 1^st^ and 4^th^ block, after breakfast and lunch time. Abbreviations: SG, sleepy group; AG, alert group; AMIF-HF, Auto-mutual information function in high frequency band; mean RR, mean RR interval; MWT, maintenance of wakefulness test; MSLT, multiple sleep latency test.

Differences between groups occurred exclusively during the MSLT (Table 3). We found that AMIF (in Total and HF band) and CORR (in Total Band) showed a more regular RR rhythm in the SG than in the AG (*p*<0.004, after Bonferroni correction). This behaviour was confirmed in each of 5 MSLT naps throughout the day (p<0.004 after Bonferroni correction). During the MWT, the RR rhythm was similar in both groups. Differences between nap tests mainly occurred in the SG, showing a more regular RR rhythm during MSLT than during MWT in AMIF (in all frequency bands, *p*-range <0.001–0.002) and CORR (in Total band, p<0.001). In the AG, no differences were observed between MWT and MSLT except for the AMIF in HF band, which showed an increased regularity of the RR rhythm during the MSLT (p<0.001). Fig 1(A) shows the evolution of AMIF in HF band in both groups throughout the whole nap protocol.

**Table 3:** Linear, Time-Frequency Representation and Nonlinear measures at the beginning of MSLT and MWT: descriptive data and differences between groups.

		MSLT			MWT			
		SG	AG	*p*-value	SG	AG	*p*	*p-*value
**LINEAR MEASURES**	**MeanRRi (ms)**	985,23 ± 149,61	964,39 ± 94,85	NS (0,915)	937,90 ± 138,16	921,32 ± 97,26		NS (0,796)
	**STDRRi (ms)**	47,22 ± 18,69	36,29 ± 10,16	NS (0,091)	40,91 ± 18,52	38,56 ± 10,61		NS (0,819)
	**LF (a.u)**	58,25 ± 11,85	56,98 ± 7,35	NS (0,532)	56,71 ± 10,00	57,56 ± 8,58		NS (0,915)
	**HF (a.u)**	41,75 ± 11,85	43,02 ± 7,35	NS (0,532)	43,29 ± 10,00	42,44 ± 8,58		NS (0,915)
	**LF/HF ratio (a.u)**	1,87 ± 0,88	1,73 ± 0,73	NS (0,615)	1,65 ± 0,71	1,82 ± 0,65		NS (0,410)
**TFR MEASURES**	**TFR-TB (ms** ^**2**^ **)**	4582,56 ± 3914,34	2624,44 ± 1398,56	NS (0,156)	4417,47 ± 4366,42	3126,50 ± 2300,46		NS (0,522)
	**TFR-LF (a.u)**	1,75E-05 ± 7,22E-06	1,48E-05 ± 2,82E-06	NS (0,532)	1,52E-05 ± 3,49E-06	1,36E-05 ± 2,69E-06		NS (0,120)
	**TFR-HF (a.u)**	8,29E-06 ± 4,97E-06	5,79E-06 ± 1,76E-06	NS (0,065)	5,35E-06 ± 1,71E-06	4,90E-06 ± 1,99E-06		NS (0,353)
**NON-LINEAR MEASURES**	**AMIF-TB (a.u)**	0,39 ± 0,04	0,35 ± 0,03	0,001*	0,36 ± 0,04	0,35 ± 0,02		NS (0,855)
	**AMIF-LF (a.u)**	0,48 ± 0,04	0,44 ± 0,03	NS (0,004)	0,46 ± 0,04	0,45 ± 0,03		NS (0,474)
	**AMIF-HF (a.u)**	0,34 ± 0,04	0,32 ± 0,02	0,001*	0,32 ± 0,02	0,30 ± 0,02		NS (0,075)
	**CORR-TB (a.u)**	0,36 ± 0,05	0,31 ± 0,04	0,001*	0,30 ± 0,04	0,31 ± 0,07		NS (0,749)
	**CORR-LF (a.u)**	0,28 ± 0,05	0,27 ± 0,03	NS (0,419)	0,25 ± 0,04	0,26 ± 0,04		NS (0,512)
	**CORR-HF (a.u)**	0,36 ± 0,09	0,33 ± 0,05	NS (0,532)	0,34 ± 0,06	0,32 ± 0,06		NS (0,139)

Results are expressed as mean ± SD. After Bonferroni correction, level of significance was *: p<0.004. NS, non-significant.

Abbreviations: MSLT, multiple sleep latency test; MWT, maintenance of wakefulness test; SG, Sleepy Group; AG, Alert Group; meanRRi, mean RR interval; SDVRRi, standard deviation of the RR interval; LF(nu), low-frequency spectral power; HF(nu) high-frequency spectral power; LF/HF ratio, low-frequency to high-frequency spectral power ratio; TFR measures, Time-frequency representation in: total band (TFR-TB), low-frequency band (TFR-LF), high-frequency band (TFR-HF); Auto-mutual information function (AMIF) in: total band (AMIF-TB), low-frequency band (AMIF-LF), high-frequency band (AMIF-HF); Correntropy (CORR) in: total band (CORR-TB); low-frequency band (CORR-LF); high-frequency band. Units of measurement: ms, milliseconds; a.u, absolute units; ms^2^, square milliseconds.

No differences between groups were observed in any traditional linear and TFR measures, either in MSLT or MWT. However, we found that mean RR interval was longer (i.e. slower heart rate) during the MSLT than during the MWT, independently of the sleepiness group: the SG (985.2 ± 149.6 ms and 937.9 ± 138.2 ms, *p*<0.001) and the AG (964.4 ± 94.9 ms and 921.3 ± 97.3 ms, *p*<0.001). Fig 1(B) shows the evolution of mean RR interval in both groups throughout the nap protocol.

**Fig 2:**
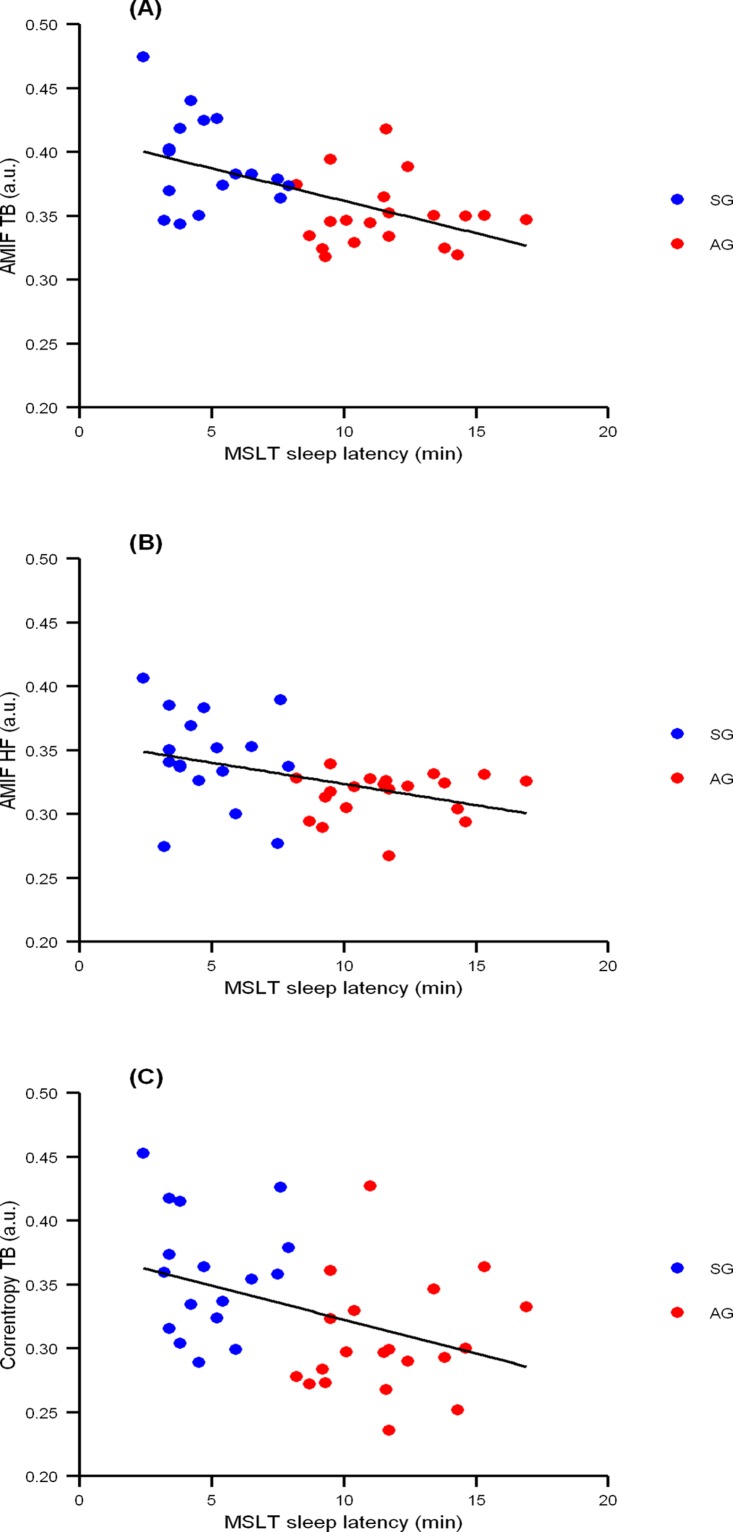
Correlation between AMIF-TB (A), AMIF-HF (B), and CORR-TB (C) with sleep latency from MSLT of all subjects, when using averages. Sleepy group, in blue, alert group, in red. Regression lines are represented in black. Note that patients with shorter sleep latencies showed an increased regularity of the RR rhythm in (A) *rho* -0.47, (B) *rho* -0.49 and (C) *rho* -0.41 (all *p*<0.05). Abbreviations: Auto-mutual information function (AMIF) in: total band (AMIF-TB), high-frequency band (AMIF-HF); Correntropy function in total band (CORR-TB); MSLT, Multiple Sleep Latency Test.

Correlation analysis showed that patients with shorter MSLT sleep latency had higher regularity of the RR rhythm. The best correlations were found with AMIF in HF band (*rho* -0.49, *p* = 0.002) and AMIF in Total band (*rho* -0.47, *p* = 0.003), followed by CORR in Total band (*rho* -0.41, *p* = 0.01) (Fig 2).

Each of the three measures yielded a *Sen* ≥70%, *Spe* >75% and *AUC* >0.80 discriminating the AG from the SG. However, AMIF in Total and HF bands achieved slightly better results than CORR in Total band (Table 4).

**Table 4:** Discrimination between the SG and AG at the MSLT.

	Sen (%)	Spe (%)	AUC
**AMIF-TB**	71	80	0,83
**AMIF-HF**	76	85	0,81
**CORR-TB**	71	75	0,81

Abbreviations: SG, Sleepy Group; AG, Alert Group; MSLT, multiple sleep latency test; Sen, sensitivity; Spe, specificity, AUC, area under the curve; Auto-mutual information function (AMIF) in: total band (AMIF-TB),) and high-frequency band (AMIF-HF); Correntropy in total band (CORR-TB).

## Discussion

To our knowledge, this is the first study that uses linear and non-linear measures applied to RR signal in order to detect SDB patients with and without objective EDS. Using this approach we have demonstrated that the regularity of the RR rhythm during the first 3-min of wakefulness at the MSLT allows differentiating the sleepy from the alert patients. In contrast, in a situation where sleep latencies were much longer as occurs during the MWT, non-linear dynamics did not differ between groups.

We have observed that both AMIF (in HF and Total band) and CORR (in Total band) functions showed an increased regularity of the RR rhythm in sleepy patients, in comparison to alert patients during the first wakefulness period at the beginning of the MSLT. In a previous study, Melia U et Al. evaluated the regularity (conversely, the complexity) of the EEG signal using CORR functions and found that sleepy patients had a more regular EEG signal (analyzed in the *β* band) in the occipital region than alert patients also during the same nap test [24]. The findings obtained with the analysis of RR interval go in the same direction than those from the EEG, suggesting a common mechanism. We hypothesize that the increased regularity observed in the SG during the first waking 3-min could reflect the autonomic changes occurring with the proximity of sleep onset. The SG had shorter sleep latencies than the AG (4.4 ± 1.96 minutes *versus* 11.66 ± 2.41 minutes, respectively) and, therefore, even at the beginning of the test they were much closer to the EEG sleep onset than the AG. We confirmed these association with the correlation analysis showing that the RR signal was more regular when MSLT sleep latency was shorter (i.e. when sleep onset was closer). The lack of differences between groups at the MWT may also support our hypothesis. During this test, patients are instructed to remain awake and sleep latencies are expected to be longer, as we observed in our study (MWT sleep latencies were longer than 10 minutes in both groups). However, we cannot exclude other factors that characterize MWT, such as the body position, the open eyes or the environmental dim-light that could have conditioned our results during this test.

We failed to observe differences between groups in traditional linear and TFR measures. Our results contrast with a study performed in healthy young adults that showed an increased heart rate and a higher sympatho-vagal balance in the alert subjects during MSLT (MSLT sleep latency <7min), compared to the sleepy subjects (MSLT sleep latency <7min) [22]. However, the sample, subject‘s age, nap protocol and window size of our work differ from that study and may have influenced the results. In another study performed in SDB patients with and without EDS during nocturnal sleep, Lombardi et al. [11] showed that the sleepy group had an increased cardiac sympatho-vagal balance (low frequency to high frequency spectral power ratio) throughout the whole night. We assumed that during wakefulness, confounding factors that may influence the cardiac rhythm such as sleep-related respiratory events are not to be expected. However, we cannot discard that breathing instability typical of the transition from wakefulness to sleep may have occurred in some patients during the measurement of HRV and, therefore, conditioned the results. Another work by Donadio et al. [13], who evaluated the muscle sympathetic nerve activity by microneurography, determined that severity of EDS in sleepy SDB patients was related to daytime sympathetic hyperactivity. In our study, we failed to find similar results due in part to the different protocols and techniques used to measure ANS activity, since Donadio et al. had not an alert SDB group to compare with.

Despite the mean RR internal was unable to differentiate between the AG and SG, it varied between MSLT and MWT regardless the level of sleepiness. The mean RR interval was reliably shorter (i.e. heart rate was faster) during the MWT, without associated changes in other traditional linear measures. It has been argued that the differences between tests are related to changes in physiological level of arousal. In fact, specific methodological conditions distinguish each test, promoting to remain awake in MWT and to fall asleep in MSLT. From this point of view, the effect of upright tilt-up [33, 34] and its combination with the instruction to remain awake could explain the increase in heart rate, accompanying the longer sleep latencies typically observed during the MWT [21]. We have also corroborated the influence of the time of day on heart rate, with special emphasis during the digestion in the 4^th^ block of MSLT/MWT [21]. At that moment, heart rate was almost identical for MSLT and MWT in both groups and achieved its highest values of all day (i.e. lowest RR interval) (see Fig 1(B)).

This study has several inherent limitations. First, we could not analyze HRV with the recommended 5-min windows of RR signal because of the nature of our investigation [14]. The SG, which was characterized by very short periods of wakefulness before the appearance of sleep onset, showed sleep latencies shorter than 5 minutes in 65 of 100 MSLT naps that prevented the calculation of mean HRV values in this group and the comparison with the AG. The selection of window size of 3-min increased the number of available naps to evaluate HRV but, again, the most somnolent patients did not show enough RR signal in 40 of 100 MSLT naps. Therefore, we decided to find a compromise between the window length analyzed and the number of naps excluded from the analysis. To maximize the number of available MSLT naps in the SG, we included those naps with sleep latencies between 2 and 3 minutes and we evaluated HRV with a window size that equaled the length of sleep latency. In this way, we could get more information about the HRV associated to sleepiness in the SG and thus, to calculate the mean HRV values and compare the groups. A second limitation is that we did not monitor breathing during naps and thus, we cannot exclude the inclusion of occasional windows containing sleep-related respiratory events that could have influenced our results. This cannot be, however, a major problem because the MWT and MSLT protocols that we used were ended as soon as the patients fall asleep. Finally, care must be taken with the discrimination ability of the AMIF and CORR functions to identify each group, considering the small number of subjects and the lack of validation set.

There is a clear need for a simple and practical tool that could be routinely administered during wakefulness to diagnose those individuals with EDS and to prevent the undesirable consequences related to EDS. In the clinical practice, MSLT is considered the reference test for objectively measure daytime sleepiness and is mainly based on EEG. However, it requires more than 10 electrodes correctly placed on the scalp and face and a trained technician to interpret the signals. In our study, we have evaluated EDS with a simpler an easier to record signal than EEG. Two EKG derivations and a 3-min window of waking RR signal recorded at the beginning of MSLT were enough to detect significant differences in regularity of cardiac rhythm between the AG and the SG. De Gennaro et al. have also measured the oculomotor activity (another easy to record biological signal) during the first waking 150-sec from MSLT and found that a decrease in spontaneous blinking and an increase in slow eye movements were associated with shorter sleep latencies [35]. These two works reflect the potential applicability of alternative biological signals to EEG for monitoring sleepiness in the clinical practice and diagnose EDS. Furthermore, the reliable detection of drowsiness in real-life scenarios such as driving has received increased interest in the last few decades, mainly for the purpose of preventing driving accidents and errors [36]. Although several automatic detection methods exist, those that employ biological signal processing are the most feasible because they inform about the body’s response to drowsiness. Some high-risk professions such as professional drivers could benefit from these automatic detectors of sleepiness for preventing accidents at the wheel. We propose that the development of new EKG indexes based on AMIF and correntropy functions may allow the automatic detection of sleepiness in this setting. However, we are still far and further studies should be addressed.

In conclusion, non-linear dynamics of the RR rhythm may detect those SDB patients with and without EDS before the appearance of MSLT sleep onset. Larger studies including different degrees of daytime sleepiness, and different window sizes of RR signal analysis would be of interest to elucidate the importance of non-linear measures of HRV in the identification of EDS while the subject is awake. The evaluation along the entire wake-sleep transition during sleep latency tests and also during other scenarios (i.e. driving simulations) should also be tested to elucidate if the findings observed at the beginning of the test would remain stable or changed as sleep onset approaches.

## Supporting Information

S1 File Calculation of Time-Frequency Representation, Auto-Mutual Information Function and Correntropy function. (PDF)
