# Supplementary material for: Regularity of Cardiac Rhythm as a Marker of Sleepiness in Sleep Disordered Breathing
Source: PLoS One. 2015 Apr 10;10(4):e0122645. doi: 10.1371/journal.pone.0122645 (PMC4393025; doi:10.1371/journal.pone.0122645)
Supplement: S1 File — (PDF) [file pone.0122645.s001.pdf]

Supporting information File

1. Time Frequency Representation

Time Frequency Representation based on Choi-Williams distribution (CWD) (1) is calculated by convoluting the Wigner distribution (WD) (2) and the Choi-Williams (CW) exponential (3) [1]. CWD removes the interference terms present in the Wigner distribution, and is thus used here to clearly characterize the dominant frequency components. Moreover, it has a very good resolution in both the time and frequency domains.

$$T_{RR}(t,f) = \iint_{-\infty}^{\infty} h(t-t',f-f')\, W_{RR}\,(t',f')dt'df' \tag{1}$$

$$W_{RR}(t,f) = \int_{-\infty}^{\infty} RR(t+\tau/2)\ RR^{*}(t-\tau/2)e^{-j2\pi f\tau}d\tau \tag{2}$$

$$h(t,f) = \sqrt{\frac{4\pi}{\sigma_c}}e^{-4\pi^2\frac{(tf)^2}{\sigma_c}} \tag{3}$$

where σ_c was set to 0.005 using the estimation criterion proposed in a previous study [2]. Using this value of σ_c the interferences produced in the time-frequency plane by using sinusoidal functions, located in the RR frequency bands of LF and HF are eliminated. In order to minimize the boundary effect, each segment $RR(t+\tau/2)$ $RR^{*}(t-\tau/2)$ was multiplied by a Hanning window with length 256 samples.

The functions, instantaneous power (*IP*) and instantaneous frequency (*IF*), were obtained from $T_{RR}(t,f)$, and the spectrum was divided into the characteristic frequency

bands of the *RR*(*t*): VLF, <0.04 Hz; LF, 0.04-0.15 Hz; HF, 0.15-0.4 Hz; TB, total-frequency band. *IP* was calculated as the area under the curve of the $T_{RR}(t,f)$ at each instant. The normalized powers LF and HF and the rate LF/HF were calculated. *IF* was defined as the mean frequency of the spectrum at each instant [2]. The mean value (*m*) was calculated on the *IF* functions with respect to the time.

2. Auto-mutual information function

AMIF is a metric to estimate both linear and nonlinear dependences between two time series [3,4], x_t and $x_{t+\tau}$. It can be regarded as a nonlinear equivalent of the correlation function. AMIF is calculated by the distribution of the probability amplitudes of $x_t = RR(t)$ and $x_{t+\tau} = RR(t+\tau)$, and the joint probability of these time series, based on Shannon entropy.

$$AMIF\_Sh(\tau) = \sum_{x_t,\, x_{t+\tau}} P\{x_t, x_{t+\tau}\}\, log_2\left(\frac{P\{x_t, x_{t+\tau}\}}{P\{x_t\}P\{x_{t+\tau}\}}\right) \qquad (4)$$

Probabilities and joint probabilities were computed on the basis of a quantization in 5 bits. This function describes how the information of a signal (AMIF value at τ=0) decreases over a prediction time intervals (AMIF values at τ>0). Increasing information loss is related to decreasing predictability, and increasing complexity of the signal [5].

AMIF was also defined from Rényi entropy as in (5).

$$AMIF\_Re_q(\tau) = \frac{1}{q-1} log_2 \sum_{x_t,\, x_{t+\tau}} \frac{P^q\{x_t, x_{t+\tau}\}}{P^{q-1}\{x_t\}P^{q-1}\{x_{t+\tau}\}} \qquad (5)$$

In equation 5, the largest probabilities most influence the $AMIF\_Re_q$ when q>1 and the smallest probabilities most influence the values of $AMIF\_Re_q$ when 0<q<1. The

$AMIF\_Re_q$ converge to the Shannon AMIF when $q \rightarrow 1$. In this work, AMIF was calculated using a discrete time delay $0 \leq \tau \leq 100$ samples for different values of the control parameter of Rényi entropy: q = {0.1, 0.2, 0.5, 2, 3, 5, 10, 30, 50, 100} and $q \rightarrow 1$ using (4). AMIF was normalized by its maximum value that corresponds to $\tau=0$.

3. Correntropy function

CORR is a similarity measure of signals that generalizes the autocorrelation function to nonlinear spaces.

$$\hat{V}(\tau) = \frac{1}{N}\sum_{t=1}^{N} \kappa(x(t) - x(t-\tau)) \quad (6)$$

κ is a symmetric positive kernel function, defined by the Gaussian kernel:

$$\kappa = \frac{1}{\sqrt{2\pi}\sigma} e^{-\frac{(RR(t)-RR(t-\tau))^2}{2\sigma^2}} \quad (7)$$

where σ is the size of the kernel determined in this study by Silverman's rule [6] of density estimation:

$$\sigma = 0.9\, A\, N^{-1/5} \quad (8)$$

In this rule, A is the smaller value between the standard deviation of the data samples and data interquartile range scaled by 1.34 and N is the number of samples. A powerful advantage of the CORR function is its robustness against impulsive noise. This advantage is due to the fact that when an outlier is present, the inner product in the feature space computed via the Gaussian kernel tends to be zero.

In this work, CORR was calculated using a discrete time delay $0 \leq \tau \leq 100$ samples.

4. Defined measures from AMIF and CORR

AMIF and CORR were applied to *RR*(*t*) time series (TB) and to *RR*(*t*) filtered in the frequency bands: VLF, <0.04 Hz; LF, 0.04-0.15 Hz; HF, 0.15-0.4 Hz. In order to quantify and extract the essential information contained on AMIF and CORR, the mean value and the first relative maximum were calculated. In this way, the values of the AMIF and CORR measures describe the complexity of the RR time series: higher values of *m* and *maxL* are associated with less complexity, more regularity of the RR signal.

In the following table, the notation used in the main text is associated with the calculated measures of AMIF and CORR:

Table 1. Notation of AMIF and CORR measures

MAIN TEXT NOTATION	MEASURE
**AMIF-TB (a.u)**	Mean value of AMIF_Re: q=2 in TB
**AMIF-LF (a.u)**	Mean value of AMIF_Re: q=2 in LF
**AMIF-HF (a.u)**	Mean value of AMIF_Re: q=2 in HF
**CORR-TB (a.u)**	First relative maximum of CORR in TB
**CORR-LF (a.u)**	First relative maximum of CORR in LF
**CORR-HF (a.u)**	First relative maximum of CORR in HF

5. References

1. Cohen L. Time-frequency analysis. Prentice Hall Signal Processing Series; 1995.
2. Clariá F, Vallverdú M, Riba J, Romero S, Barbanoj MJ, Caminal P. Characterization of the cerebral activity by time-frequency representation of evoked EEG potentials. Physiol Meas. 2011;32:1327-46.
3. Plattard D, Soret M, Troccaz J, Vassal P, Giraud JY, Champleboux G et al. Patient setup using portal images: 2D/2D image registration using mutual information. Computer Aided Surgery. 2000;5:246–62.
4. Wells WM 3rd, Viola P, Atsumi H, Nakajima S, Kikinis R. Multi-modal volume registration by maximization of mutual information. Med Image Anal. 1996;1:35-51.
5. Jeong J, Gore JC, Peterson BS. Mutual information analysis of the EEG in patients with Alzheimer's disease. Clin Neurophysiol. 2001;112:827–35
6. Santamaria I, Pokharel PP, Principe JC. Generalized correlation function: definition, properties and application to blind equalization. IEEE Transactions on Signal Processing. 2006;54:2187–97.
